# 2-Chloro-*N*-chloro­methyl-*N*-(2-ethyl-6-methyl­phen­yl)acetamide

**DOI:** 10.1107/S1600536808011902

**Published:** 2008-05-03

**Authors:** Zu-Wei Song

**Affiliations:** a College of Science, Qingdao Agricultural University, Qingdao 266109, People’s Republic of China

## Abstract

The title compound, C_12_H_15_Cl_2_NO, was synthesized as an inter­mediate for the synthesis of the herbicide Acetochlor. The crystal structure exhibits weak inter­molecular C—H⋯O hydrogen bonds, which link the mol­ecules into zigzag chains along the *b* axis.

## Related literature

For details of the biological activities of Acetochlor, see: Breaux (1986 ▶). For bond-length data, see: Allen *et al.* (1987 ▶).
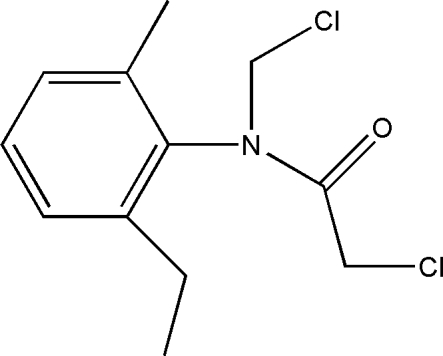


## Experimental

### 

#### Crystal data

C_12_H_15_Cl_2_NO
*M* *_r_* = 260.15
Orthorhombic, 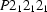

*a* = 8.3012 (17) Å
*b* = 9.3787 (19) Å
*c* = 16.575 (3) Å
*V* = 1290.4 (5) Å^3^
*Z* = 4
Mo *K*α radiation
μ = 0.48 mm^−1^
*T* = 296 (2) K
0.33 × 0.27 × 0.17 mm

#### Data collection

Rigaku R-AXIS RAPID IP area-detector diffractometer
Absorption correction: multi-scan (*ABSCOR*; Higashi, 1995 ▶) *T*_min_ = 0.857, *T*_max_ = 0.922
20457 measured reflections
2403 independent reflections
1506 reflections with *I* > 2σ(*I*)
*R*_int_ = 0.051

#### Refinement

*R*[*F* ^2^ > 2σ(*F* ^2^)] = 0.036
*wR*(*F* ^2^) = 0.075
*S* = 0.77
2403 reflections
145 parameters
H-atom parameters constrained
Δρ_max_ = 0.21 e Å^−3^
Δρ_min_ = −0.14 e Å^−3^
Absolute structure: Flack (1983 ▶), 691 Friedel pairs
Flack parameter: 0.00 (9)

### 

Data collection: *RAPID-AUTO* (Rigaku, 2004 ▶); cell refinement: *RAPID-AUTO*; data reduction: *RAPID-AUTO*; program(s) used to solve structure: *SHELXTL* (Sheldrick, 2008 ▶); program(s) used to refine structure: *SHELXTL*; molecular graphics: *SHELXTL*; software used to prepare material for publication: *SHELXTL*.

## Supplementary Material

Crystal structure: contains datablocks I, global. DOI: 10.1107/S1600536808011902/cv2400sup1.cif

Structure factors: contains datablocks I. DOI: 10.1107/S1600536808011902/cv2400Isup2.hkl

Additional supplementary materials:  crystallographic information; 3D view; checkCIF report

## Figures and Tables

**Table 1:** Hydrogen-bond geometry (Å, °)

*D*—H⋯*A*	*D*—H	H⋯*A*	*D*⋯*A*	*D*—H⋯*A*
C12—H12*B*⋯O1^i^	0.97	2.43	3.375 (4)	164

Symmetry code: (i) 
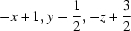
.

**Fig. 1.**
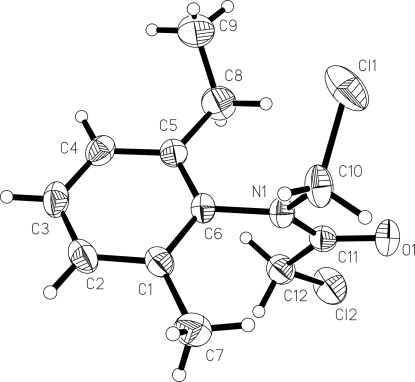
The molecular structure of (I), with atom labels and 30% probability displacement ellipsoids for non-H atoms.

## supplementary crystallographic information

### Comment

Acetochlor is an herbicide developed by Monsanto and Zeneca. It is a member of the class of herbicides known as chloroacetanilides. Its mode of action is elongase inhibition, and inhibition of geranylgeranyl pyrophosphate (GGPP) cyclization enzymes, part of the gibberellin pathway (Breaux, 1986). It is used to control weeds in corn, and is particularly useful as a replacement for atrazine in the case of some important weeds. The title compound, (I), was synthesized as an intermediate for the synthesis of Acetochlor. We report here the crystal structure of (I).

In (I) (Fig. 1), all bond lengths and angles are normal (Allen *et al.*, 1987). The mean plane N1/O1/C6/C10/C11 (with largest deviation of 0.036 (2) Å) and benzene ring C1-C6 form a dihedral angle of 78.0 (3)°. The crystal packing exhibits weak intermolecular C–H···O hydrogen bonds (Table 1), which link the molecules into zigzag chains along *b* axis.

### Experimental

The xylene solution containing *N*-methylene-2'-methyl-6'-ethyl-aniline was introduced into a mixture of 1.2 g (0.01 mol) of chloroacetyl chloride and 2 g xylene at 293 K to 313 K under continuous stirring. After about 15 minutes of stirring, 2.5 g of dry ethanol were introduced into mixture at 293 K to 313 K. The reaction mixture was stirred for 5 h, whereupon accoholysis proceeded. At the end of the reaction, 6 g of water were introduced into the mixture, and the phases were separated. The upper organic phase was washed acid-free with about 10 g of water,and the xylene solution, containing about 2.5 g of the desired end product, was separated. Single crystals suitable for X-ray measurements were obtained by recrystallization from ethanol and dichloromethane at room temperature.

### Refinement

H atoms were positioned geometrically (C—H = 0.93–0.97 Å), and refined using a riding model, with *U*_iso_(H) = 1.2 (1.5 for methyl groups) times *U*_eq_(C).

### Crystal data

**Table:**

C_12_H_15_Cl_2_NO	*F*_000_ = 544
*M**_r_* = 260.15	*D*_x_ = 1.339 Mg m^−^^3^
Orthorhombic, *P*2_1_2_1_2_1_	Mo *K*α radiation λ = 0.71073 Å
Hall symbol: P 2ac 2ab	Cell parameters from 987 reflections
*a* = 8.3012 (17) Å	θ = 2.2–27.5º
*b* = 9.3787 (19) Å	µ = 0.48 mm^−^^1^
*c* = 16.575 (3) Å	*T* = 296 (2) K
*V* = 1290.4 (5) Å^3^	Plate, colorless
*Z* = 4	0.33 × 0.27 × 0.17 mm

### Data collection

**Table:**

Rigaku R-AXIS RAPID IP area-detector diffractometer	2403 independent reflections
Radiation source: rotating anode	1506 reflections with *I* > 2σ(*I*)
Monochromator: graphite	*R*_int_ = 0.051
*T* = 296(2) K	θ_max_ = 25.5º
ω scans at fixed χ = 45°	θ_min_ = 2.5º
Absorption correction: multi-scan(ABSCOR; Higashi, 1995)	*h* = −10→10
*T*_min_ = 0.857, *T*_max_ = 0.923	*k* = −11→11
20457 measured reflections	*l* = −19→20

### Refinement

**Table:**

Refinement on *F*^2^	Hydrogen site location: inferred from neighbouring sites
Least-squares matrix: full	H-atom parameters constrained
*R*[*F*^2^ > 2σ(*F*^2^)] = 0.037	*w* = 1/[σ^2^(*F*_o_^2^) + (0.0172*P*)^2^] where *P* = (*F*_o_^2^ + 2*F*_c_^2^)/3
*wR*(*F*^2^) = 0.075	(Δ/σ)_max_ = 0.001
*S* = 0.77	Δρ_max_ = 0.21 e Å^−^^3^
2403 reflections	Δρ_min_ = −0.14 e Å^−^^3^
145 parameters	Extinction correction: none
Primary atom site location: structure-invariant direct methods	Absolute structure: Flack (1983), 691 Friedel pairs
Secondary atom site location: difference Fourier map	Flack parameter: 0.00 (9)

### Special details

**Table:**

Geometry. All e.s.d.'s (except the e.s.d. in the dihedral angle between two l.s. planes) are estimated using the full covariance matrix. The cell e.s.d.'s are taken into account individually in the estimation of e.s.d.'s in distances, angles and torsion angles; correlations between e.s.d.'s in cell parameters are only used when they are defined by crystal symmetry. An approximate (isotropic) treatment of cell e.s.d.'s is used for estimating e.s.d.'s involving l.s. planes.
Refinement. Refinement of *F*^2^ against ALL reflections. The weighted *R*-factor *wR* and goodness of fit *S* are based on *F*^2^, conventional *R*-factors *R* are based on *F*, with *F* set to zero for negative *F*^2^. The threshold expression of *F*^2^ > σ(*F*^2^) is used only for calculating *R*-factors(gt) *etc*. and is not relevant to the choice of reflections for refinement. *R*-factors based on *F*^2^ are statistically about twice as large as those based on *F*, and *R*- factors based on ALL data will be even larger.

### Fractional atomic coordinates and isotropic or equivalent isotropic displacement parameters (Å^2^)

**Table:**

	*x*	*y*	*z*	*U*_iso_*/*U*_eq_	
Cl1	0.86248 (17)	1.10317 (8)	0.59963 (5)	0.0986 (4)	
Cl2	0.61827 (15)	0.78116 (9)	0.88510 (5)	0.0859 (3)	
O1	0.6020 (3)	0.9889 (2)	0.75349 (11)	0.0616 (6)	
N1	0.7273 (3)	0.8602 (2)	0.65690 (13)	0.0444 (6)	
C1	0.7314 (4)	0.6328 (3)	0.58464 (17)	0.0495 (7)	
C2	0.8149 (4)	0.5122 (3)	0.56042 (17)	0.0605 (9)	
H2A	0.7634	0.4437	0.5291	0.073*	
C3	0.9741 (5)	0.4929 (3)	0.58248 (18)	0.0611 (9)	
H3A	1.0287	0.4116	0.5656	0.073*	
C4	1.0523 (4)	0.5913 (3)	0.62872 (17)	0.0558 (8)	
H4A	1.1592	0.5761	0.6432	0.067*	
C5	0.9737 (4)	0.7147 (3)	0.65459 (16)	0.0468 (7)	
C6	0.8129 (3)	0.7325 (3)	0.63222 (14)	0.0417 (7)	
C7	0.5558 (4)	0.6500 (4)	0.56248 (19)	0.0717 (10)	
H7A	0.5165	0.7390	0.5832	0.108*	
H7B	0.4947	0.5730	0.5853	0.108*	
H7C	0.5447	0.6488	0.5048	0.108*	
C8	1.0590 (4)	0.8198 (4)	0.7098 (2)	0.0742 (11)	
H8A	0.9981	0.9080	0.7100	0.089*	
H8B	1.0563	0.7819	0.7643	0.089*	
C10	0.7080 (4)	0.9714 (3)	0.59945 (18)	0.0631 (9)	
H10A	0.6057	1.0181	0.6094	0.076*	
H10B	0.7029	0.9292	0.5461	0.076*	
C11	0.6641 (3)	0.8780 (3)	0.73246 (16)	0.0456 (7)	
C12	0.6724 (4)	0.7457 (3)	0.78494 (15)	0.0586 (8)	
H12A	0.7812	0.7080	0.7837	0.070*	
H12B	0.6009	0.6736	0.7631	0.070*	
C9	1.2274 (5)	0.8537 (5)	0.6897 (3)	0.145 (2)	
H9A	1.2684	0.9222	0.7275	0.217*	
H9B	1.2325	0.8926	0.6362	0.217*	
H9C	1.2911	0.7684	0.6923	0.217*	

### Atomic displacement parameters (Å^2^)

**Table:**

	*U*^11^	*U*^22^	*U*^33^	*U*^12^	*U*^13^	*U*^23^
Cl1	0.1649 (10)	0.0562 (5)	0.0746 (6)	−0.0264 (7)	0.0055 (7)	0.0077 (4)
Cl2	0.1260 (8)	0.0752 (5)	0.0565 (5)	−0.0120 (6)	0.0273 (6)	−0.0026 (4)
O1	0.0704 (15)	0.0524 (12)	0.0621 (12)	0.0181 (13)	0.0044 (12)	−0.0097 (10)
N1	0.0505 (15)	0.0416 (13)	0.0412 (13)	0.0070 (12)	−0.0038 (11)	−0.0019 (11)
C1	0.060 (2)	0.0421 (16)	0.0461 (16)	−0.0048 (16)	0.0026 (15)	−0.0013 (14)
C2	0.086 (3)	0.0459 (18)	0.0501 (17)	−0.0116 (19)	0.0115 (18)	−0.0087 (14)
C3	0.078 (3)	0.0427 (17)	0.063 (2)	0.0139 (19)	0.019 (2)	0.0035 (16)
C4	0.051 (2)	0.0562 (18)	0.0601 (19)	0.0094 (17)	0.0075 (16)	0.0121 (16)
C5	0.052 (2)	0.0452 (16)	0.0431 (15)	−0.0001 (15)	0.0030 (14)	0.0026 (14)
C6	0.0493 (19)	0.0379 (15)	0.0380 (14)	0.0062 (14)	0.0014 (13)	0.0006 (13)
C7	0.065 (2)	0.078 (2)	0.072 (2)	−0.0091 (19)	−0.0055 (19)	−0.0107 (18)
C8	0.063 (3)	0.077 (2)	0.083 (3)	0.0028 (19)	−0.023 (2)	−0.002 (2)
C10	0.084 (2)	0.0505 (17)	0.0543 (18)	0.0211 (17)	−0.0101 (18)	−0.0019 (15)
C11	0.0418 (18)	0.0460 (17)	0.0491 (16)	−0.0023 (15)	−0.0055 (14)	−0.0043 (14)
C12	0.069 (2)	0.0537 (17)	0.0531 (17)	−0.0057 (17)	0.0113 (16)	−0.0039 (15)
C9	0.058 (3)	0.104 (3)	0.272 (7)	−0.018 (3)	0.017 (4)	−0.060 (4)

### Geometric parameters (Å, °)

**Table:**

Cl1—C10	1.781 (3)	C5—C8	1.521 (4)
Cl2—C12	1.752 (3)	C7—H7A	0.9600
O1—C11	1.212 (3)	C7—H7B	0.9600
N1—C11	1.368 (3)	C7—H7C	0.9600
N1—C10	1.421 (3)	C8—C9	1.472 (5)
N1—C6	1.451 (3)	C8—H8A	0.9700
C1—C2	1.387 (4)	C8—H8B	0.9700
C1—C6	1.398 (4)	C10—H10A	0.9700
C1—C7	1.511 (4)	C10—H10B	0.9700
C2—C3	1.384 (4)	C11—C12	1.517 (4)
C2—H2A	0.9300	C12—H12A	0.9700
C3—C4	1.364 (4)	C12—H12B	0.9700
C3—H3A	0.9300	C9—H9A	0.9600
C4—C5	1.396 (4)	C9—H9B	0.9600
C4—H4A	0.9300	C9—H9C	0.9600
C5—C6	1.395 (4)		
			
C11—N1—C10	118.7 (2)	C9—C8—C5	116.5 (3)
C11—N1—C6	123.1 (2)	C9—C8—H8A	108.2
C10—N1—C6	118.2 (2)	C5—C8—H8A	108.2
C2—C1—C6	117.8 (3)	C9—C8—H8B	108.2
C2—C1—C7	119.9 (3)	C5—C8—H8B	108.2
C6—C1—C7	122.2 (3)	H8A—C8—H8B	107.3
C3—C2—C1	120.5 (3)	N1—C10—Cl1	115.3 (2)
C3—C2—H2A	119.7	N1—C10—H10A	108.5
C1—C2—H2A	119.7	Cl1—C10—H10A	108.5
C4—C3—C2	121.0 (3)	N1—C10—H10B	108.5
C4—C3—H3A	119.5	Cl1—C10—H10B	108.5
C2—C3—H3A	119.5	H10A—C10—H10B	107.5
C3—C4—C5	120.7 (3)	O1—C11—N1	122.1 (2)
C3—C4—H4A	119.6	O1—C11—C12	123.8 (3)
C5—C4—H4A	119.6	N1—C11—C12	114.1 (2)
C6—C5—C4	117.7 (3)	C11—C12—Cl2	112.13 (19)
C6—C5—C8	121.9 (3)	C11—C12—H12A	109.2
C4—C5—C8	120.3 (3)	Cl2—C12—H12A	109.2
C5—C6—C1	122.2 (3)	C11—C12—H12B	109.2
C5—C6—N1	119.5 (3)	Cl2—C12—H12B	109.2
C1—C6—N1	118.3 (2)	H12A—C12—H12B	107.9
C1—C7—H7A	109.5	C8—C9—H9A	109.5
C1—C7—H7B	109.5	C8—C9—H9B	109.5
H7A—C7—H7B	109.5	H9A—C9—H9B	109.5
C1—C7—H7C	109.5	C8—C9—H9C	109.5
H7A—C7—H7C	109.5	H9A—C9—H9C	109.5
H7B—C7—H7C	109.5	H9B—C9—H9C	109.5
			
C6—C1—C2—C3	−0.5 (4)	C11—N1—C6—C5	−79.7 (3)
C7—C1—C2—C3	−177.9 (3)	C10—N1—C6—C5	100.6 (3)
C1—C2—C3—C4	0.3 (5)	C11—N1—C6—C1	102.0 (3)
C2—C3—C4—C5	−0.4 (4)	C10—N1—C6—C1	−77.7 (3)
C3—C4—C5—C6	0.6 (4)	C6—C5—C8—C9	−140.5 (4)
C3—C4—C5—C8	176.7 (3)	C4—C5—C8—C9	43.6 (5)
C4—C5—C6—C1	−0.9 (4)	C11—N1—C10—Cl1	88.9 (3)
C8—C5—C6—C1	−176.9 (3)	C6—N1—C10—Cl1	−91.4 (3)
C4—C5—C6—N1	−179.1 (2)	C10—N1—C11—O1	−5.1 (4)
C8—C5—C6—N1	4.9 (4)	C6—N1—C11—O1	175.2 (3)
C2—C1—C6—C5	0.8 (4)	C10—N1—C11—C12	172.3 (3)
C7—C1—C6—C5	178.1 (3)	C6—N1—C11—C12	−7.4 (4)
C2—C1—C6—N1	179.1 (2)	O1—C11—C12—Cl2	−11.6 (4)
C7—C1—C6—N1	−3.6 (4)	N1—C11—C12—Cl2	171.1 (2)

### Hydrogen-bond geometry (Å, °)

**Table:**

*D*—H···*A*	*D*—H	H···*A*	*D*···*A*	*D*—H···*A*
C12—H12B···O1^i^	0.97	2.43	3.375 (4)	164

Symmetry codes: (i) −*x*+1, *y*−1/2, −*z*+3/2.
